# Assessing Gene Expression Related to Cisplatin Resistance in Human Oral Squamous Cell Carcinoma Cell Lines

**DOI:** 10.3390/ph15060704

**Published:** 2022-06-03

**Authors:** Hyeong Sim Choi, Young-Kyun Kim, Pil-Young Yun

**Affiliations:** 1 Department of Oral and Maxillofacial Surgery, Section of Dentistry, Seoul National University Bundang Hospital, 82 Gumi-ro 173 beon-gil, Bundang-gu, Seongnam 13620, Korea; r2013@snubh.org (H.S.C.); kyk0505@snubh.org (Y.-K.K.); 2 Department of Dentistry and Dental Research Institute, School of Dentistry, Seoul National University, 101 Daehak-ro, Jongno-gu, Seoul 03080, Korea

**Keywords:** oral squamous cell carcinoma, cisplatin, RNA-Seq, cell adhesion molecules, proteoglycans

## Abstract

Cisplatin-based chemotherapy has been effectively used to treat oral cancer, but treatment often fails owing to the development of drug resistance. However, the important gene expression alterations associated with these resistances remain unclear. In this study, we aimed to identify the gene expressions related to cisplatin resistance in oral squamous cell carcinoma (OSCC) cell lines. RNA samples were obtained from three cisplatin-resistant (YD-8/CIS, YD-9/CIS, and YD-38/CIS) and -sensitive (YD-8, YD-9, and YD-38) cell lines. Global gene expression was analyzed using RNA sequencing (RNA-Seq). Differentially expressed genes were determined. Based on the gene ontology (GO) and Kyoto Encyclopedia of Genes and Genomes (KEGG) databases, functional enrichment and signaling pathways analyses were performed. Candidate genes selected from RNA-Seq analysis were validated by quantitative real-time polymerase chain reaction (qRT-PCR) analysis. The YD-8/CIS and YD-9/CIS samples had very similar expression patterns. qRT-PCR analysis was performed on selected genes commonly expressed between the two samples. The expression levels of 11 genes were changed in cisplatin-resistant samples compared with their parental samples; several of these genes were related to cell adhesion molecules and proteoglycans in cancer pathways. Our data provide candidate genes associated with cisplatin resistance in OSCC, but further study is required to determine which genes have an important role. Nevertheless, these results may provide new ideas to improve the clinical therapeutic outcomes of OSCC.

## 1. Introduction

In 2020, there were 377,713 new cases of oral cancer and 177,757 deaths worldwide “Lip, oral cavity. Available online: http://gco.iarc.fr/today/fact-sheets-cancers (accessed on 05 January 2022)” [1]. Oral cancer is approximately twice as common in men as in women [2,3]. It is well known that several factors, including smoking, drinking alcohol, and chewing areca nut, contribute to the initiation of oral cancer [2,3,4]. The term oral cancer tends to be used interchangeably with oral squamous cell carcinoma (OSCC), as most oral cancers are squamous cell carcinoma [5,6,7]. Similar to many other cancer treatments, OSCC has three main treatment options: surgery, radiotherapy, and chemotherapy. This study focuses on chemotherapy [7]. Chemotherapy is beneficial for many patients with cancer, and cisplatin is one of the most widely used first-line drugs to treat OSCC [8,9]. Cisplatin was studied for its potential tumor-suppressing effect in the 1960s, becoming the first FDA-approved platinum compound for cancer treatment in 1978 [9,10]. Despite its therapeutic benefits, its use as chemotherapy is often limited by the development of cisplatin resistance [6,11,12]. Although the patient’s initial response to cisplatin-based chemotherapy is usually good, the clinical efficacy is significantly reduced when cisplatin resistance occurs [6,9]. Current knowledge has suggested several factors that are associated with resistance to platinum-based drugs in OSCC: DNA damage response, epigenetic mechanisms, programmed cell death, tumor microenvironment, transport process, epithelial-mesenchymal transition (EMT), and cancer stem cells [9]. However, there have been few studies on the associated gene expression alterations [9]. For example, ERCC1, a DNA repair-related gene that leads to cisplatin resistance via snail-mediated upregulation and ATP binding cassette (ABC) transporters, which act as pumps to lower intracellular drug levels, were overexpressed in cisplatin-resistant cells [9,13,14]. Hence, it is necessary to investigate the genetic mechanisms of cisplatin resistance as a means to determine new therapeutic approaches to overcome this resistance. In our previous studies, we established three cisplatin-resistant cell models with progressively acquired chemoresistance derived from three cisplatin-sensitive cell lines to elucidate the mechanism of cisplatin resistance in OSCC [13,15,16]. From these results, we confirmed that some genes related to ABC transporters or EMT are associated with cisplatin resistance, but further research was needed to identify changes in the expression of more genes. In this study, we tried to determine the differences in gene expression patterns that may provide an indication of the mechanism of resistance and to clarify their role in cisplatin-resistant cell lines (YD-8/CIS, YD-9/CIS, and YD-38/CIS) established from existing oral cancer cell lines (YD-8, YD-9, and YD-38) by using RNA-Seq and qRT-PCR analysis.

## 2. Results

### 2.1. Seauencing and Unigene Assembly

An RNA-Seq approach was used to assess the differential expression across the transcriptome in cisplatin-resistant OSCC cells (YD-8/CIS, YD-9/CIS, and YD-38/CIS) and their parental cells. We sequenced a total of 48,790,084 reads in YD-8, 54,379,620 reads in YD-8/CIS, 49,933,926 reads in YD-9, 54,914,214 reads in YD-9/CIS, 49,258,722 reads in YD-38, and 54,601,488 reads in YD-38/CIS (Table 1). After read cleaning, there were 47,574,638, 53,296,460, 48,870,688, 53,564,371, 47,880,336, and 53,489,836 read pairs in YD-8, YD-8/CIS, YD-9, YD-9/CIS, YD-38, and YD-38/CIS samples, respectively (Table 1). The number of mapped reads ranged from 40 to 47 million (Table 1). The mapping of RNA-Seq reads to the reference genome was successful, with a mapping rate of 89.2%, 88.4%, 83.7%, 87.3%, 86.4%, and 78.8% for the YD-8, YD-8/CIS, YD-9, YD-9/CIS, YD-38, and YD-38/CIS samples, respectively (Table 1). We obtained 41,494,996 uniquely mapped reads in YD-8 (a mapping rate of 87.2%), 46,142,562 uniquely mapped reads in YD-8/CIS (a mapping rate of 86.6%), 39,607,099 uniquely mapped reads in YD-9 (a mapping rate of 81.0%), 45,748,506 uniquely mapped reads in YD-9/CIS (a mapping rate of 85.4%), 40,323,662 uniquely mapped reads in YD-38 (a mapping rate of 84.2%), and 41,168,565 uniquely mapped reads in YD-38/CIS (a mapping rate of 77.0%) (Table 1).

### 2.2. Analysis of Differentially Expressed Genes (DEGs)

**Figure 1:**
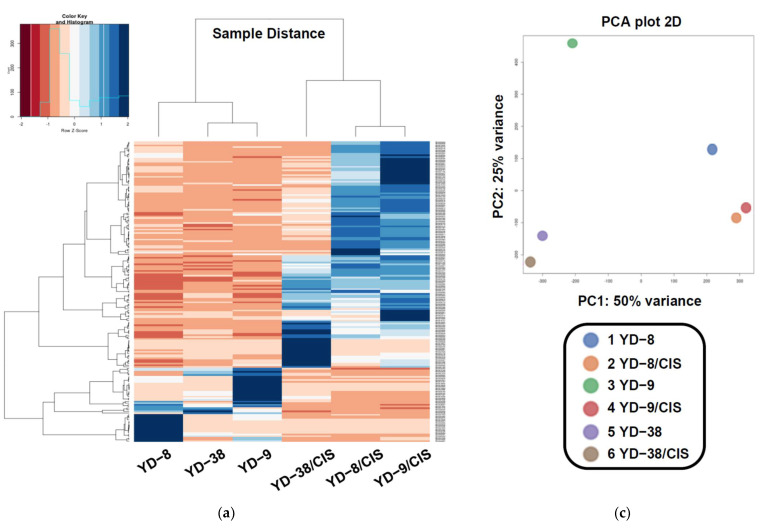

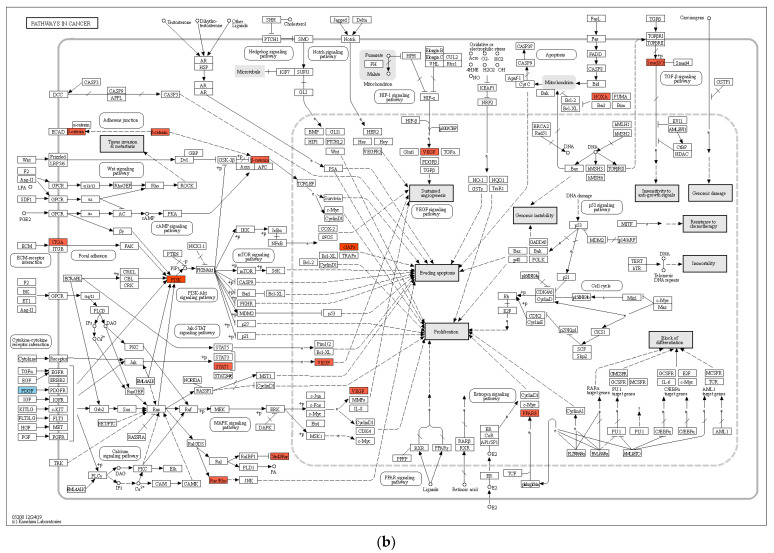
Heatmap clustering, KEGG pathway, and PCA analysis for all the RNA-Seq samples. (**a**) The heatmap of the distance matrix obtained with the DESeq2 package on regularized-logarithm transformed counts showing an overview of similarities and dissimilarities between the RNA-Seq samples. The heatmap shows three distinct groups of cells (YD-8 + YD-8/CIS, YD-9 + YD-9/CIS, and YD-38 + YD-38/CIS) and within each group, there were two subgroups of cells (cisplatin-resistant cells and parental cells). Red indicates upregulated genes and blue indicates downregulated genes (*q*-value < 0.05). (**b**) Expression of genes in cancer pathways in parental samples and cisplatin-resistant samples. Genes in the colored box have significantly different expression. Red indicates upregulated genes and blue indicates downregulated genes. (**c**) PCA plot of the overall gene expression, showing the separation of YD-8/CIS and YD-9/CIS cells from other cells. PCA is based on the abundances of all transcripts detected in the RNA-Seq analysis. The color legend is reported at the bottom of the plot.

To evaluate the global gene expression in all the samples, we produced a distance matrix heatmap and performed hierarchical cluster analysis using the data from 18,777 genes (Figure 1a). Sample distances were investigated to assess similarity and dissimilarity between samples. The results of the heatmap clustering analysis showed that the YD-9 sample was relatively closer to the YD-38 sample than to the YD-8 sample. In the results of the three cisplatin-resistant cell samples, the YD-8/CIS and YD-9/CIS samples showed the highest correlation in gene expression levels, whereas the YD-38/CIS sample had the lowest correlation with the other two samples.

We investigated the potential pathways of cisplatin-resistant samples by performing the KEGG database analysis between parental and cisplatin-resistant samples (Figure 1b). Pathway analysis was performed on genes that were significantly upregulated or downregulated in cisplatin-resistant cells (YD-8/CIS, YD-9/CIS, and YD-38/CIS) compared to the parental cells (YD-8, YD-9, and YD-38). All 12 genes in the cancer pathway were significantly altered: 11 were upregulated in YD-8/CIS and YD-9/CIS cells and 1 was downregulated.

Next, principal component analysis (PCA) was used to gain insight into how different the RNA-Seq samples were, by differentiating the samples based on their eigenvalues of gene expression (Figure 1c). The PCA plot provides a visualization of the variance between samples by generating a two-dimensional plot for all samples. As expected, the variance of each sample was 50%. In the comparison between the YD-8 and YD-9 samples, there was a large dispersion, suggesting that each sample had a different gene expression pattern. In contrast, the YD-8/CIS vs. YD-9/CIS samples were relatively less dispersed, meaning that the genes in both samples may have more similar expression patterns. The correlation of gene expression between the two samples (shown and normalized transcripts per million (TPM)), represented based on the scale of Pearson’s correlation coefficient in Table 2, is presented in Table 3. As revealed in the correlation table, the correlation coefficient between YD-8/CIS and YD-9/CIS was 0.960, which indicates that the two samples are very highly correlated. These results suggested that YD-8 and YD-9 cell lines displayed very similar genetic expression after acquiring resistance to cisplatin.

### 2.3. Functional Enrichment and Pathway Analyses of DEGs

**Figure 2:**
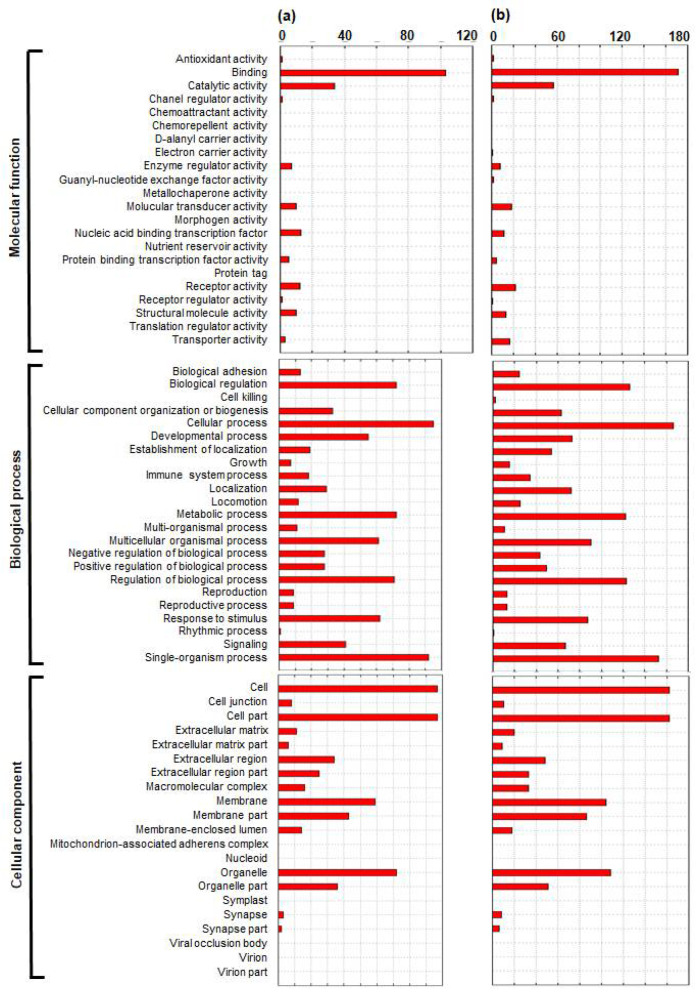
Histogram of gene ontology (GO) classification. GO functional enrichment analysis of differentially expressed gene (DEGs) (**a**) in YD-8/CIS relative to YD-8 and (**b**) in YD-9/CIS relative to YD-9. The functions of genes identified in three main classifications: molecular function, biological process, and cellular component. The *x*-axis represents the number of genes and the *y*-axis represents the ontology categories.

To investigate the biological significance of putative DEGs identified while comparing each group (YD-8 vs. YD-8/CIS and YD-9 vs. YD-9/CIS), we tested against the background set of all the GO-annotated genes in Metascape to obtain statistically significant differentially expressed transcripts with *p* values of <0.001 (Figure 2a,b). The GO terms identified were distributed in biological process (BP) (22 GO terms), cellular component (CC) (23 GO terms), and molecular function (MF) (21 GO terms). The two groups had similar GO classifications. In total, 36 statistically significant different GO terms were found for the YD-8 vs. YD-8/CIS comparison, and distributed in MF (four GO terms), BP (twenty-five GO terms), and CC (seven GO terms). In total, 17 statistically significant different GO terms were discovered in the YD-9 vs. YD-9/CIS comparison, and distributed in MF (two GO terms), BP (nine GO terms), and CC (six GO terms).

**Figure 3:**
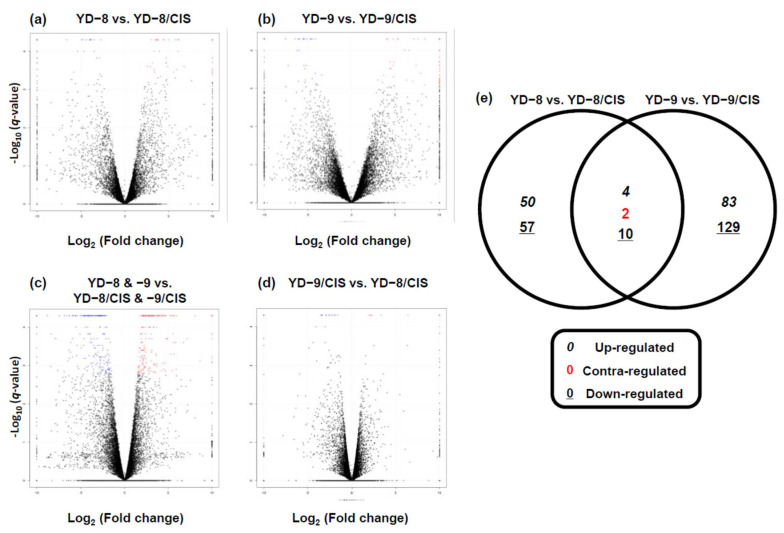
Volcano plots and Venn diagrams of expressed genes. Volcano plot and scatter plot for differentially expressed genes in YD-8 vs. YD-8/CIS (**a**), YD-9 vs. YD-9/CIS (**b**), YD-8 and YD-9 vs. YD-8/CIS and YD-9/CIS (**c**), and YD-9/CIS vs. YD-8/CIS (**d**). In the volcano plots, the *X*-axis is value of fold change and the *Y*-axis is *q*-value, which indicates the significance level of the expression difference. The red dots represent significantly upregulated genes with at least two-fold change and the blue dots represent significantly downregulated genes with at least two-fold change. (**e**) Venn diagrams showing the number of differentially expressed transcripts (*p*-value < 0.05) in the YD-8 and YD-9 samples relative to the cisplatin-resistant samples (YD-8 vs. YD-8/CIS and YD-9 vs. YD-9/CIS datasets). The Venn diagram was made using an online tool “Calculate and draw custom Venn diagrams. Available online: http://bioinformatics.psb.ugent.be/webtools/Venn/ (accessed on 5 November 2021)”.

Using a minimum log2 fold change of 2 and a maximum −log10 adjusted *q*-value of 0.05 as the cutoffs, genes with significant differences in expression between the two groups were selected and presented on a volcano plot (Figure 3a–d). The gene expression in the comparison of YD-8 and YD-9 against YD-8/CIS and YD-9/CIS revealed that 55 genes and 88 genes were significantly upregulated and 68 and 140 genes were downregulated, respectively (Figure 3a,b and Table 4). In the YD-8/CIS and YD-9/CIS samples, the number of genes significantly upregulated or downregulated compared with YD-8 and YD-9 was 188 and 242, respectively (Figure 3c and Table 4). Compared with the YD-9/CIS sample, the number of genes significantly upregulated or downregulated in the YD-8/CIS sample was 12 and 23 (Figure 3d and Table 4). We observed little difference when comparing gene expression in the YD-8/CIS and YD-9/CIS samples (Figure 3d). The red dots represent upregulated genes (>2.0 fold change) and the blue dots represent downregulated genes (>2.0 fold change). In Figure 3e, Venn diagrams are presented of the up- and downregulated genes in the YD-8 vs. YD-8/CIS and YD-9 vs. YD-9/CIS comparisons shown in Table 4. We found that 16 overlapping DEG genes were associated with the YD-8 vs. YD-8/CIS group and the YD-9 vs. YD-9/CIS group; of these, four genes were upregulated, ten genes were downregulated, and two genes were contra-regulated (Figure 3e).

**Figure 4:**
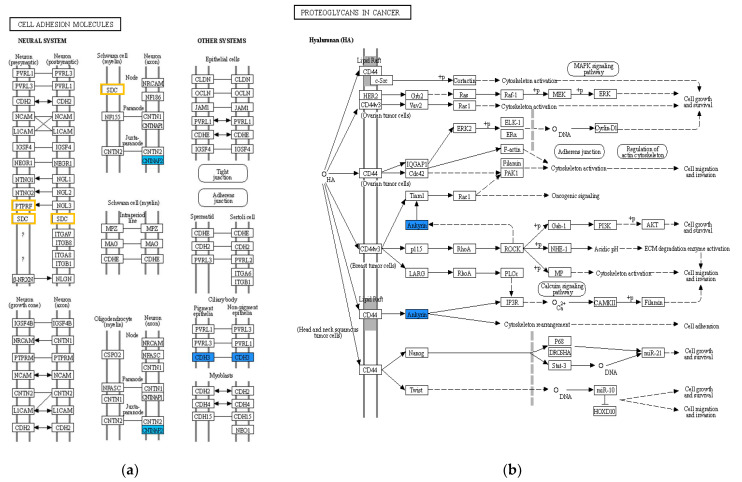
KEGG pathway for 16 genes. (**a**) Cell adhesion molecules and (**b**) proteoglycans in cancer pathways for the YD-8 vs. YD-8/CIS and YD-9 vs. YD-9/CIS comparisons. Genes in the color box have significantly different expression. Red indicates upregulated genes, blue indicates downregulated genes, and yellow indicates contra-regulated genes.

The names of the previously selected 16 genes and the information on which pathways each gene belongs to are listed in Table 5. These genes were found to be included in 13 pathways (Table 6). We also analyzed these 16 genes using the KEGG database and found that, as in our previous data, there were changes in the cell adhesion molecule pathway (Figure 4a) and the proteoglycans in cancer pathway (Figure 4b). The results showed that the expression of ANK3 (in the proteoglycans in cancer pathway), CDH3, and CNTNAP2 (in the cell adhesion molecule pathway) was downregulated. As revealed in our findings, YD-8/CIS had a very similar expression pattern to YD-9/CIS.

### 2.4. Validation of RNA-Seq Results by qRT-PCR

**Figure 5:**
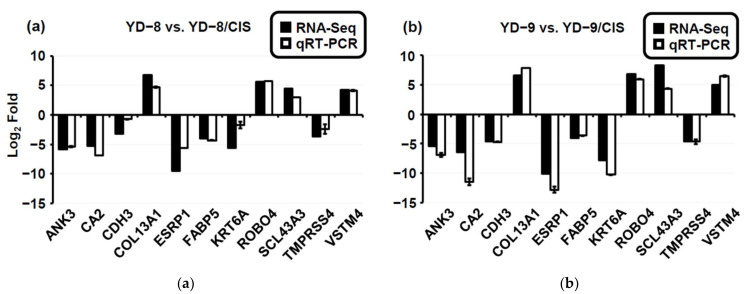
Relative fold change for 11 genes. (**a**,**b**) Comparison of relative fold change between RNA-Seq and qRT-PCR results in parental (YD-8 and YD-9) and cisplatin-resistant (YD-8/CIS and YD-9/CIS) cell lines (*p* < 0.001). In the RNA-Seq results, differentially expressed genes are shown as a black bar. Differentially expressed genes confirmed by qRT-PCR are shown as a white bar. Data are represented as mean values ± SD, *n* = 3 compared with that in the YD-8 or YD-9 sample, *p* < 0.01. If log2 of gene expression was below the mean of log2 across the panel of genes, the genes were defined as downregulated; conversely, upregulated genes had values log2 above the mean.

Some genes had significantly different expression in both the YD-8/CIS and YD-9/CIS samples compared to their parental samples. To validate the reliability of the expression profiles identified using the RNA-Seq and DEG analysis, we selected 11 candidate genes for qRT-PCR analysis, excluding the two contra-regulated genes (LPHN2 and SDC2) and three other genes (GRHL2, CNTNAP2 and AUTS2) with significantly lower expression rates in the RNA-Seq analysis data. As expected, similar correlations of the relative change values between the RNA-Seq data and qRT-PCR data are shown in Figure 5a,b. We identified four significantly upregulated genes (SLC43A3, VSTM4, COL13A1, and ROBO4) and seven downregulated genes (CDH3, ESRP1, CA2, FABP5, KRT6A, ANK3, and TMPRSS4). These results indicated that the RNA-Seq data reliably identified potential genes in cisplatin-resistant OSCC cells.

## 3. Discussion

In our previous study, we generated three cisplatin-resistant oral cancer cell lines and established that MDR- and EMT-related molecules were overexpressed compared to the relevant parental cell line. In these cisplatin-resistant cell lines, it was also found that the combination of cisplatin and paclitaxel inhibited the induction of apoptosis by paclitaxel alone, and that the combination of cisplatin and cetuximab inhibited cell migration and proliferation. However, as the gene expression profiles of these six cell lines were previously unknown, in this study, they were investigated using RNA-Seq.

The heatmap analysis showed that the samples were divided into two groups with different gene expression patterns for the parental and cisplatin-resistant samples. Moreover, from the KEGG database analysis, several genes were overexpressed in the cisplatin-resistant samples compared to their parental samples, especially in the pathways of cancer. From the heatmap and correlation of gene expression results, it was confirmed that gene expression patterns of YD-8/CIS and YD-9/CIS, except for YD-38/CIS, were changed very similarly among our three cisplatin-resistant samples. Therefore, we focused on identifying which genes were changed in the cisplatin-resistant samples (YD-8/CIS and YD-9/CIS) compared with each parental sample (YD-8 and YD-9) when resistance to cisplatin was acquired using other analyses.

GO analysis revealed that the number of genes in each ontology category was generally similar in the MF, BP, and CC classifications. In the volcano plot analysis of several comparison groups of four samples (YD-8, YD-9, YD-8/CIS, and YD-9/CIS), it was also shown that the YD-8/CIS and YD-9/CIS groups were the most similar. Therefore, we constructed a Venn diagram to find genes with similar expression changes in YD-8/CIS and YD-9/CIS samples compared with each parental sample; of the 16 genes, four were upregulated, two were contra-regulated, and ten were downregulated.

Then, we analyzed the pathways with these genes and were particularly interested in the downregulation of the two pathways (hsa04514-cell adhesion molecules and hsa05205-proteoglycans in cancer). Cell adhesion molecules (CAMs) help cells to attach to other cells or extracellular matrices (ECMs) in a process called cell adhesion [17,18]. CAMs are usually divided into five groups, and the cadherin family is a member of one of these groups [19]. Proteoglycans are also considered as one of the CAM classes [20]. Many proteoglycans and cadherins in the tumor microenvironment are known to play important roles that influence the biology of various types of cancer, including proliferation, adhesion, angiogenesis, metastasis, and influencing tumor progression [17,18,21,22,23]. Four genes (SDC2, CNTNAP2, CDH3, and ANK3) were included in these two pathways, but SDC2 was a contra-regulated gene and CNTNAP2 was excluded owing to the low difference in expression. It is well known that a reduction in CDH3 and/or ANK3 expression is a common malignant event in OSCC progression; thus, it is interesting that these genes are downregulated in our established cisplatin-resistant cell lines.

Furthermore, we tried to compare the difference in the expression of the 11 previously selected genes with the results of RNA-Seq analysis through qRT-PCR analysis. Subsequently, it was confirmed that all 11 genes followed similar expression patterns: seven genes were downregulated and four genes were upregulated. Although our previous studies revealed that EMT characteristics were increased in the three cisplatin-resistant OSCC cell lines, it was unclear which genes were the key regulators. The EMT is a process in which epithelial cells lose their characteristics such as cell–cell adhesion and polarity maintenance, and are converted into mesenchymal cells to exhibit migratory behavior and invasiveness [24]. Recently, the EMT has attracted attention as a process that contributes to chemoresistance [24,25]. Overexpression of CHD3 in OSCC has been reported to be associated with poor prognosis as well as resistance to cisplatin [26]. In addition, a previous study found that hyaluronan binding promotes multidrug resistance gene 1 expression, cytoskeletal protein (ankyrin)-induced drug fluxes, and chemoresistance in cancer stem cells and tumor progression [27]. Therefore, this study has provided evidence that these genes may play an important role in the EMT when OSCC cell lines have acquired resistance to cisplatin. However, we could not clearly understand the mechanism by which genes increase resistance to cisplatin in OSCC cell lines, and this study may be insufficient to represent overall resistance to cisplatin because of the small number of samples or lack of clinical validation. Therefore, further study is needed to elucidate the relationship between these genes (including CDH3 and ANK3) and cancer metastasis in oral cancer cells and tissue.

## 4. Materials and Methods

### 4.1. Reagents

Cisplatin (PubChem CID: 84691; PubChem, Bethesda, MD, USA) was purchased from JW Medical C. (Seoul, Korea) and dissolved in distilled water at 1 mg/mL. Each aliquot of the stock solution was stored at −20 °C until use.

### 4.2. Cell Lines and Cell Culture

Three human OSCC cell lines (YD-8, YD-9 and YD-38) were obtained from the Korean Cell Line Bank (Seoul, Korea). These YD cell lines were from the tongue (YD-8), buccal cheek (YD-9), and lower gingiva (YD-38) [28]. Their cisplatin-resistant cell lines (YD-8/CIS, YD-9/CIS, and YD-38/CIS) were developed as described previously [13]. In brief, their parental cell lines were exposed to increasing concentrations of cisplatin, starting at 0.1 μg/mL and ending at 2 μg/mL. If there was a large amount of cell death among cisplatin-sensitive parental cells during cisplatin treatment, the cultures were maintained in normal medium until the surviving cells restored a normal growth pattern, and then the cisplatin concentration was intermittently increased. These cell lines were maintained in RPMI-1640 medium (Biowest, Nuaillé, France) containing 10% heat-inactivated fetal bovine serum (Biowest, Nuaillé, France) and 1% penicillin–streptomycin (Biowest, Nuaillé, France) at 37 °C and supplied with 5% CO_2_. The cisplatin-resistant cell lines were continuously maintained with 2 μg/mL of cisplatin. In our previous studies, we confirmed the establishment and characterization of three cisplatin-resistant cell lines [13,15,16].

### 4.3. RNA Preparation

Total RNA was extracted from cells collected in a 1.5 mL tube using an RNA-spin™ Total RNA Extraction Kit (iNtRON Biotechnology, Inc., Seongnam, Korea) in accordance with the manufacturer’s instructions. Briefly, cells were harvested by centrifugation and the supernatant was discarded. R-buffer was added to the pellet and mixed well. An equal amount of 70% ethanol was added to the R-buffer, loaded onto the column, and the flow-through was discarded after centrifuging. Washing buffer A was added to wash the column and the flow-through was discarded after centrifuging. In the same way, washing buffer B was added to wash the column. After the membrane was dried, RNA was extracted by adding elution buffer. The concentration and purity of the isolated RNA were measured using the NanoDrop™ 2000 spectrophotometer (Thermo Fisher Scientific Ins., Waltham, MA, USA).

### 4.4. Generation of the Transcriptome Library and RNA Sequencing

Total RNA integrity was checked using an Agilent 2100 BioAnalyzer (Agilent Technologies, Santa Clara, CA, USA) for an RNA Integrity Number greater than 6. The following were determined by YD-8 (9.9), YD-8/CIS (10.0), YD-9 (8.3), YD-9/CIS (10.0), YD-38 (10.0), and YD-38/CIS (9.9). We prepared the libraries for 100 bp paired-end sequencing using a TruSeq Stranded mRNA Sample Preparation Kit (Illumina, San Diego, CA, USA). Namely, mRNA molecules were purified and fragmented from 2 μg of total RNA using oligo-dT-attached magnetic beads. The fragmented mRNAs were synthesized as single-stranded cDNAs through random hexamer priming. By applying this as a template for second-strand synthesis, double-stranded cDNA was prepared. After the sequential processes of end repair, A-tailing, and adapter ligation, cDNA libraries were amplified by PCR. The quality of these cDNA libraries was evaluated using the Agilent 2100 BioAnalyzer (Agilent, Santa Clara, CA, USA). The libraries were quantified using the KAPA library quantification kit (Kapa Biosystems, Wilmington, MA, USA) in accordance with the manufacturer’s library quantification protocol. Following the cluster amplification of denatured templates, sequencing was progressed as paired-end (2 × 100 bp) using the Illumina platform sequencer (Illumina, San Diego, CA, USA).

### 4.5. Analysis of RNA Sequence Reads and Sequence Alignment

Low-quality reads were filtered using the following criteria: reads containing more than 10% of skipped bases (marked as “N”s); reads containing more than 40% of bases with quality scores less than 20; and reads for which the average quality score of each read was less than 20. The whole filtering process was performed using the in-house scripts. Filtered reads were mapped to the reference genome related to the species using the TopHat aligner [29].

### 4.6. Gene Expression Estimation

Gene expression level was measured with Cufflinks v2.1.1 (Cole Trapnell’s lab, Washington, D.C., USA) using the gene annotation database for the appropriate species [30]. To improve the accuracy of the measurement, multiread-correction and frag-bias-correct options were applied. All other options were set to default values.

### 4.7. Heatmap

A heat map (or heatmap) is a data visualization technique that uses color and hierarchical clustering to identify patterns in data. It is commonly used to visualize RNA-Seq results and helps to visualize gene expression patterns throughout the samples. It not only shows the data values but also allows a natural visual pattern to be created by using dark colors when the data values are high or large, and light colors when the data values are low or small. In brief, heatmaps are very effective visualization charts to pattern what a large amount of data suggest, even without specific numbers.

### 4.8. PCA

PCA is a distance-based ordination technique to reduce the dimensionality of the dataset by transforming to a new set of variables (the principal components) to summarize the features of the data. The technique is very useful for the visualization and analysis of data, including determining the presence of a correlation between data points.

### 4.9. Correlation Analysis

To determine the relationship between each sample, we performed pairwise gene correlation analysis using methods such as the Pearson, Spearman, and Kendall correlation statistics. The strength of the relationship among variables was determined based on Table 2 with the correlation results shown in Table 3.

### 4.10. DEG Analysis

DEGs were determined using Cuffdiff [31]. To enhance the accuracy of the analysis, multiread-correction and frag-bias-correct options were applied. All other options were set to default values. DEGs were identified based on the q-value threshold of less than 0.05 for correcting errors caused by multiple testing [32]. To perform the enrichment analysis, the functional enrichment tool DAVID was applied. Gene set enrichment analysis is a method to identify classes of genes or proteins that are overrepresented in a large set of genes or proteins and may have an association with disease phenotypes [33]. The method uses statistical approaches to identify significantly enriched or depleted groups of genes. The analysis provides information about the function of genes and the functional annotations of GO, which allows users to describe a gene/gene product in detail by considering three main aspects: its MF, the BP in which it participates, and its CC [34]. The GO terms were considered for 16 genes obtained from the volcano plot (4 upregulated genes, 2 contra-regulated genes, and 10 downregulated genes). To characterize the genes identified from the DEG analysis, GO-based trend analysis was performed using Fisher’s exact test [35]. In the pathway analysis using the KEGG database “KEGG Mapper − Color. Available online: https://www.genome.jp/kegg/mapper/color.html (accessed on 6 November 2021), we examined pathways in which genes were significantly changed.

### 4.11. Experimental Validation via qRT-PCR

cDNA was directly synthesized from the total cellular RNA (1 μg) using the High-Capacity cDNA Reverse Transcription Kit (Thermo Fisher Scientific, Waltham, MA, USA). Several genes of interest, including CDH3, ESRP1, CA2, SLC43A3, FABP5, VSTM4, KRT6A, ANK3, TMPRSS4, COL13A1, and ROBO4 were selected to validate the results of RNA-Seq analysis. The expression levels of these genes were analyzed using a qPCR QuantStudio 7 Flex Real-Time PCR System (Applied Biosystems, Waltham, MA, USA) using GoTaq^®^ qPCR Master Mix (Promega, Madison, WI, USA). The following qPCR reaction conditions were used: 95 °C for 2 min for the hot-start polymerase activation step, followed by 40 cycles of denaturation at 95 °C for 15 s, annealing and extension steps at 60 °C for 1 min, and a final cooling step at 4 °C. The specific primers used in this study are detailed in Table 7. The gene expression level for each sample was calculated using the comparative CT method (2^−ΔΔCT^ method) relative to the housekeeping gene GAPDH [36].

### 4.12. Statistical Analysis

The qRT-PCR data were presented as the mean ± SD (standard deviation) (*n* = 3). The significance of the differences between the two paired groups (YD-8 vs. YD-8/CIS, YD-9 vs. YD-9/CIS, and YD-38 vs. YD-38/CIS) were analyzed using a Student’s t-test carried out in Microsoft Excel (Microsoft Corporation, Redmond, WA, USA). *p* values of less than 0.01 were considered to be statistically significant.

## 5. Conclusions

In this research, gene expression profiling (RNA-Seq and qRT-PCR) data were used to analyze the differences between parental and cisplatin-resistant OSCC cell lines. The results showed that genes, including those associated with cancer pathways, were more upregulated in the cisplatin-resistant cells compared with the parental cells. The DEG analysis confirmed that the correlation was very strong and that there were similar patterns in the comparisons between the YD-8/CIS and YD-9/CIS samples and their parental samples. Several candidate genes that might be involved in the CAMs and proteoglycans in cancer pathways were identified. Based on the altered molecular characteristics of cisplatin-resistant cell lines, we assumed that the changes in the expression of 11 genes promoted the acquisition of cisplatin resistance, inducing the development of invasive species. There is a need for further investigation into the acquisition of cisplatin resistance in oral cancer cell lines, specifically to determine if the 11 selected genes lead to changes at the molecular level, especially those involved in proliferation, adhesion, migration, and angiogenesis.

## Figures and Tables

**Table 1:** Summary statistics of the sequencing analysis of the RNA-Seq libraries.

Sample (Name)	Total Reads (Raw)	Processed Reads (Cleaned)	Mapped Reads	Mapping Rate	Uniquely Mapped	Mapping Rate
YD-8	48,790,084	47,574,638	42,418,196	89.2%	41,494,996	87.2%
YD-8/CIS	54,379,620	53,296,460	47,104,007	88.4%	46,142,562	86.6%
YD-9	49,933,926	48,870,688	40,886,333	83.7%	39,607,099	81.0%
YD-9/CIS	54,914,214	53,564,370	46,767,206	87.3%	45,748,506	85.4%
YD-38	49,258,722	47,880,336	41,381,531	86.4%	40,323,662	84.2%
YD-38/CIS	54,601,488	53,489,836	42,157,460	78.8%	41,168,565	77.0%

The columns show (from left to right): total number of reads, number of processed reads, number of mapped reads, percentage of mapping rate, number of uniquely mapped reads, and percentage of uniquely mapped reads.

**Table 2:** The scale of Pearson’s correlation coefficient.

Scale of Correlation Coefficient	Value
0.00 < r ≤ 0.70	Very low correlation
0.70 < r ≤ 0.80	Low correlation
0.80 < r ≤ 0.92	Moderate correlation
0.92 < r ≤ 1.00	Very high correlation

**Table 3:** The correlation between gene expressions of samples.

**YD-8**	**YD-8/CIS**	**YD-9**	**YD-9/CIS**	**YD-38**	**YD-38/CIS**	
	0.856	0.794	0.833	0.825	0.765	**YD-8**
		0.733	0.960	0.805	0.787	**YD-8/CIS**
			0.717	0.842	0.803	**YD-9**
				0.790	0.779	**YD-9/CIS**
					0.896	**YD-38**
						**YD-38/CIS**

Pearson correlation coefficient between FPKM of genes of samples.

**Table 4:** Differential expression between parental and cisplatin-resistant cell line libraries.

Group 1	Group 2	Genes		
		Up	Down	Sum
YD-8	YD-8/CIS	55	68	123
YD-9	YD-9/CIS	88	140	228
YD-8 & -9	YD-8/CIS & -9/CIS	188	242	430
YD-9/CIS	YD-8/CIS	12	23	35

Each comparison is run between the other paired cell lines. up = upregulated; down = downregulated; sum = up- and downregulated.

**Table 5:** List of 16 genes from the Venn diagram.

Gene Symbol	Gene Name	Pathway
ADGRL2	Adhesion G Protein-Coupled Receptor L2	
ANK3	Ankyrin 3	hsa05205
AUTS2	Autism susceptibility candidate 2	
CA2	Carbonic anhydrase II	hsa04976, hsa00910, hsa04964 hsa04966, hsa04971, hsa04972
CDH3	Cadherin 3	hsa04514
CNTNAP2	Contactin-associated protein-like 2	hsa04514
COL13A1	Collagen, type XIII, alpha 1	hsa04974
ESRP1	Epithelial Splicing Regulatory Protein 1	
FABP5	Fatty acid binding protein 5	hsa03320
GRHL2	Grainyhead-like 2	
KRT6A	Keratin 6A	
ROBO4	Roundabout guidance receptor 4	
SDC2	Syndecan 2	hsa04514, hsa05144 hsa05205, hsa05418
SLC43A3	Solute carrier family 43 member 3	
TMPRSS4	Transmembrane protease serine 4	hsa05164

**Table 6:** List of KEGG pathway entries and names.

Pathway Entry	Name
hsa00910	Nitrogen metabolism
hsa03320	PPAR signaling pathway
hsa04514	Cell adhesion molecules
hsa04964	Proximal tubule bicarbonate reclamation
hsa04966	Collecting duct acid secretion
hsa04971	Gastric acid secretion
hsa04972	Pancreatic secretion
hsa04974	Protein digestion and absorption
hsa04976	Bile secretion
hsa05144	Malaria
hsa05164	Influenza A
hsa05205	Proteoglycans in cancer
hsa05418	Fluid shear stress and atherosclerosis

**Table 7:** Primer lists of qRT-PCR (5′ -> 3′).

Gene Name	Primer Sequence		Length	References
ANK3	F	AAAGGACTGCCTCAAACAGCGG	22	Origene (Gene ID: 288)
	R	CTAAGGATGCGAAGCTCTGTCG	22	
CA2	F	CAATGGTCATGCTTTCAACG	20	Clin Cancer Res. 2005 Nov 15;11(22):8201–8207.
	R	TCCATCAAGTGAACCCCAGT	20	doi: 10.1158/1078-0432.CCR-05-0816.
CDH3	F	CCCCCAGAAGTACGAGGCCCA	20	Anat Cell Biol. 2010 Jun;43(2):110–117.
	R	ACGCCACGCTGGTGAGTTGG	21	doi: 10.5115/acb.2010.43.2.110
COL13A1	F	CAAAGGGAGAAGCAGGTGTC	20	Int J Mol Sci. 2019 Oct; 20(19): 4890.
	R	TCACTGGAGAGCCTCATTGAT	21	doi: 10.3390/ijms20194890.
ESRP1	F	TCCTGCTGTTCTGGAAAGTCG	21	Cancer Lett. 2011 Jan 1;300(1):66–78.
	R	TCCGGTCTAACTAGCACTTCGTG	23	doi: 10.1016/j.canlet.2010.09.007.
FABP5	F	GCTGATGGCAGAAAAACTCAGA	22	Oncotarget. 2018 Aug 3; 9(60): 31753–31770.
	R	CCTGATGCTGAACCAATGCA	20	doi: 10.18632/oncotarget.25878.
KRT6A	F	TCACCGTCAACCAGAGTCTC	20	Mol Med Rep. 2019 May;19(5):3477–3484.
	R	GAACCTTGTTCTGCTGCTCC	20	doi: 10.3892/mmr.2019.10055.
ROBO4	F	GACACTTGGCGTTCCACCTC	20	BMC Cancer. 2008 Dec 29;8:392.
	R	AGAGCAAGGAGCGACGACAG	20	doi: 10.1186/1471-2407-8-392.
SLC43A3	F	CACCGCCACACTCATCATAG	20	J Pharm Sci. 2020 Aug;109(8):2622–2628.
	R	GGTGTTGGCCAAATAGGTTC	20	doi: 10.1016/j.xphs.2020.04.013.
TMPRSS4	F	CCGATGTGTTCAACTGGAAG	20	Br J Cancer. 2011 Nov 8;105(10):1608–1614.
	R	GAGAAAGTGAGTGGGAACTG	20	doi: 10.1038/bjc.2011.432.
VSTM4	F	TGTCACTAGCGTGACCAGCTTG	22	Origene (Gene ID: 196740)
	R	CAGCTTCGGTTTATGGAACGTGG	23	
GAPDH	F	AATCCCATCACCATCTTCCA	20	Cell Mol Life Sci. 2016 Sep 11;73:1067–1084.
	R	TGGACTCCACGACGTACTCA	20	doi: 10.1007/s00018-015-2036-6.

ANK3: Ankyrin 3; CA2: Carbonic anhydrase II; CDH3: *p*-Cadherin; COL13A1: Collagen type XIII alpha 1 chain; ESRP1: Epithelial Splicing Regulatory Protein 1; FABP5: Fatty acid binding protein 5; KRT6A: Keratin 6A; ROBO4; Roundabout4; SLC43A3: Solute carrier family 43 member 3; TMPRSS4: Transmembrane protease serine 4; VSTM4: V-set and transmembrane domain-containing protein 4; GAPDH: Glyceraldehyde 3-phosphate dehydrogenase.

## Data Availability

All data presented during the current study are included in this published article.
